# Two new species of *Dugesia* (Platyhelminthes, Tricladida, Dugesiidae) from the tropical monsoon forest in southern China

**DOI:** 10.3897/zookeys.1059.65633

**Published:** 2021-09-08

**Authors:** Lei Wang, Jin-zi Chen, Zi-mei Dong, Guang-wen Chen, Ronald Sluys, De-zeng Liu

**Affiliations:** 1 College of Life Science, Henan Normal University, Xinxiang, 453007 Henan, China Henan Normal University Xinxiang China; 2 Naturalis Biodiversity Center, Leiden, The Netherlands Xinxiang University Xinxiang China; 3 Medical College, Xinxiang University, Xinxiang 453003, China Naturalis Biodiversity Center Leiden Netherlands

**Keywords:** Genetic distance, karyology, molecular phylogeny, monsoon forest, new species, taxonomy

## Abstract

Two new species of the genus *Dugesia* (Platyhelminthes, Tricladida, Dugesiidae) from the tropical monsoon forest in southern China are described on the basis of an integrative taxonomic study involving morphology, karyology, histology, and molecular analyses. The new species *Dugesiacircumcisa* Chen & Dong, **sp. nov.** is characterised by asymmetrical openings of the oviducts; right vas deferens opening at anterior portion of the seminal vesicle and the left one opening at mid-lateral portion of the seminal vesicle; two diaphragms in ejaculatory duct, the latter being ventrally displaced and opening at the tip of the penis papilla, which is provided with a nozzle; wide duct connecting male atrium and common atrium; chromosome complement triploid with 24 metacentric chromosomes. The other new species, *Dugesiaverrucula* Chen & Dong, **sp. nov.**, is characterised by the large size of the living worm, usually exceeding 3.5 cm in length; asymmetrical openings of the oviducts; subterminal opening of ventrally displaced ejaculatory duct; vasa deferentia symmetrically opening into the postero-lateral portion of the seminal vesicle; well-developed duct between the seminal vesicle and diaphragm; single dorsal bump near the root of the penis papilla; bursal canal with pleated wall and spacious posterior section; unstalked cocoons; chromosome complement diploid with 16 metacentric chromosomes. Inter-specific molecular distances and their positions in the phylogenetic tree reveal that *D.circumcisa* and *D.verrucula* are clearly separated from their congeners.

## Introduction

Approximately 96 species of the freshwater planarian genus *Dugesia* Girard, 1850 are distributed in a major portion of the Old World and Australia (Sluys and Riutort 2018; Song et al. 2020), with only five species being known from China, viz., *Dugesiajaponica* Ichikawa & Kawakatsu, 1964, *D.sinensis* Chen & Wang, 2015, *D.umbonata* Song & Wang, 2020, *D.semiglobosa* Chen & Dong, 2021, and *D.majuscula* Chen & Dong, 2021 (Kawakatsu et al. 1976; Chen et al. 2015; Song et al. 2020; Wang et al. 2021). Only three of these species were recorded from mainland China, since *D.semiglobosa* and *D.majuscula* are known only from Hainan Island. An interesting and equally tropical mainland region is located immediately north of Hainan Island, viz., the Guangxi Province. This part of southern China has a tropical monsoon climate and is occupied by characteristic biota, such as the Wengan Biota and Chengjiang Biota (Yuan and Zhou 1999; Hou et al. 2004). The Guangxi Province is adjacent to the Indo-Chinese peninsula and thus forms a biotic link between Southern China and mainland Southeast Asia. In this paper, we describe for the first time two new species of *Dugesia* from the tropical monsoon forest in this part of China on the basis of morphological, histological, karyological, and molecular data.

## Materials and methods

### Specimen collection and culturing

**Figure 1.**
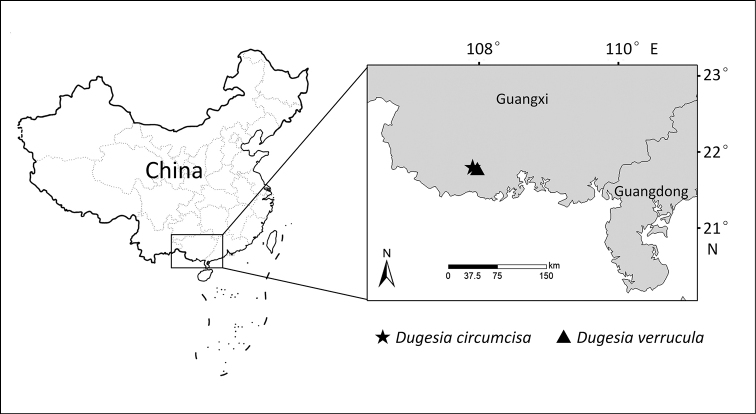
Collection sites of *Dugesia* in Guangxi Province.

On 1 January 2019, the specimens were collected in the Shiwan Dashan Mountain National Natural Reserve in Guangxi Province, where some animals were collected from under stones in a freshwater stream, while others were collected from under stones in a pond under a waterfall (for sampling localities, see Fig. 1). After collection, the worms were transferred to plastic bottles filled with spring water that during transportation to the laboratory were placed in a cooler filled with an ice bag. The planarians were cultured in autoclaved tap water at 16 °C and fed with fresh beef liver once per week. The worms were starved for at least one week before being used for karyotype and histological studies and DNA extraction. Images of their external morphology were obtained by using a digital camera attached to a stereo-microscope.

### DNA extraction, amplification, sequencing, and phylogenetic analysis

Total genomic DNA was extracted from specimens by using the QIAamp DNA Mini Tissue Kit (Qiagen, Germany), according to the manufacturer’s protocols. The primers BarS and COIR were used for amplification of fragments of the Cytochrome c oxidase subunit I (COI) (Lázaro et al. 2009; Álvarez-Presas et al. 2011). For amplification of internal transcribed spacer-1(ITS-1), the primers 9F and ITSR were used (Baguñà et al. 1999). Premix Ex Taq Hot Start Version (TaKaRa, Otsu Japan) was used for the polymerase chain reaction (PCR). Amplifications were conducted in a final volume of 30 µL under the following conditions: 5 min at 94 °C, 35 cycles of 40 s at 94 °C, an annealing step for 30 s, and 1 min at 72 °C, and 5 min at 72 °C as a final extension. The annealing temperatures were 43 °C and 45 °C, respectively, and according to those specified by Stocchino et al. (2017). Purification of PCR products and sequencing were done by GENEWIZ (Tianjin, China). Sequencing reactions were performed with the same primers used to amplify the fragments. All specimens were sequenced for both forward and reverse DNA strands. Chromatograms were visually checked. In both two new species, four specimens were used to extract DNA, from which COI and ITS-1 were amplified.

In order to determine whether the presumed new species are molecularly different from other species of *Dugesia*, we performed a phylogenetic analysis and calculated genetic distances. The ingroup included the two new species, as well as 28 other *Dugesia* species from major portions of the geographic range of the genus. *Schmidteamediterranea* (Benazzi et al., 1975) was chosen as the outgroup taxon (for GenBank accession numbers, see Table 1).

**Table 1.** GenBank accession numbers of COI and ITS-1 sequences used for molecular analyses.

Species	GenBank	
	CO I	ITS-1
* D. aethiopica *	KY498845	KY498785
* D. afromontana *	KY498846	KY498786
* D. ariadnae *	KC006972	KC007048
* D. arcadia *	KC006971	KC007044
* D. batuensis *	KF907818	KF907815
* D. benazzii *	FJ646977+FJ646933	FJ646890
* D. bengalensis *		FJ646897
* D. bifida *	KY498851	KY498791
* D. bijuga *	MH119630	
* D. circumcisa *	MZ147041	MZ146782
* D. cretica *	KC006976	KC007050
* D. deharvengi *	KF907820	KF907817
* D. elegans *	KC006984	KC007063
* D. gibberosa *	KY498857	KY498803
* D. hepta *	FJ646988+FJ646943	FJ646902
* D. japonica *	FJ646990	FJ646904
* D. majuscula *	MW533425	MW533591
* D. naiadis *	KF308756	
* D. notogaea *	FJ646993+FJ646945	FJ646908
* D. pustulata *	MH119631	
* D. ryukyuensis *	AF178311	FJ646910
* D. semiglobosa *	MW525210	MW526992
* D. sicula *	FJ646994 + FJ646947	DSU84356
* D. sigmoides *	KY498849	KY498789
* D. sinensis *	KP401592	
* D. subtentaculata *	FJ646995 +FJ646949	DSU84369
* D. umbonata *	MT176641	MT177211
* D. verrucula *	MZ147040	MZ146760
* S. mediterranea *	JF837062	AF047854

Nuclear ribosomal markers were aligned online with MAFFT (Online Version 7.247) using the G-INS-i algorithm (Katoh and Standley 2013), and were checked by using BioEdit 7.2.6.1. For aligning the protein-coding COI sequences, the TranslatorX pipeline was used (http://translatorx.co.uk; Abascal et al. 2010). Nucleotide sequences were translated into amino acid sequences, with the help of NCBI’s genetic codes table 9, followed by MAFFT (Online Version 7.247), using FFT-NS-2 progressive alignment method, checked by BioEdit 7.2.6.1, and then back-translated to nucleotide sequences. Since automated removal of gap columns and variable regions has been reported to negatively affect the accuracy of the inferred phylogeny (Dessimoz and Gil 2010; Tan et al. 2015), the Gblocks option (Talavera and Castresana 2007) was disabled. The concatenated sequences for the phylogenetic analysis were in the order ITS-1+ COI and consisted of a total of 1578 base pairs (bp), including 5.65% missing data. In the concatenated sequences, missing data were marked as “?”.

Mr Bayes v 3.2 (Ronquist et al. 2012) and RaxML 8.2.10 (Stamatakis 2014) were used to infer phylogenies with the Bayesian Inference (BI) and Maximum-likelihood (ML) method, respectively. BI was run for 3 million generations, while 25% burn-in was used under the GTR+I+G model. For the ML analysis, we performed 10,000 replicates under the GTR+I+G model. BI and ML trees were visualised and edited using Figtree v1.4.3.

The genetic distances of COI and ITS-1 were calculated by MEGA 6.06 (Tamura et al. 2013) with the Kimura 2-parameter substitution model (Lázaro et al. 2009; Solà et al. 2013).

### Histology and karyology

Histological sections were prepared as described previously by Dong et al. (2017). In brief, worms were killed and thereafter fixed in Bouin’s fluid for 24 h, and, subsequently, rinsed and stored in 70% ethanol. For histological study, specimens were dehydrated in an ascending series of ethanol solutions, after which they were cleared in xylene and embedded in synthetic paraffin. Serial sections were made at intervals of 6 μm and were stained with haematoxylin-eosin. Photomicrographs were taken with a Leica digital camera attached to a compound microscope. Histological preparations of specimens have been deposited in the Zoological Museum of the College of Life Science of Henan Normal University (**ZMHNU**), Xinxiang, China, and Naturalis Biodiversity Center, Leiden, The Netherlands (**RMNH**).

Karyological preparations were obtained by air-drying, following methods described by Dong et al. (2017). In brief, worms were cut transversally into three pieces, which were cultured in distilled water for three days. Regenerative blastemas were treated with a 0.02% colchicine solution at 13 °C for 2.5–3.5 h and then placed in 0.1% KCl hypotonic solution at 16 °C for approximately 2.0–3.5 h. Hereafter, the blastemas were washed with deionised water and then fixed on a slide for ca. 30 s in each of the following solutions: fixative fluid I (glacial acetic acid: absolute alcohol: deionised water in the ratio of 3:3:4), fixative fluid II (glacial acetic acid: absolute alcohol in the ratio of 1:1) and fixative fluid III (glacial acetic acid). Subsequently, the dispersed cells were dried at room temperature for 24 h, and stained with a 0.5% Giemsa solution for 12–15 min. The mitotic metaphase chromosomes were observed and photographed under a compound microscope equipped with a digital camera. Well-spread sets of metaphase plates from five or six randomly selected individuals were used for karyotype analysis; karyotype parameter measurements were carried out as described previously by Chen et al. (2008). Chromosomal nomenclature follows Levan et al. (1964).

## Results

### Molecular phylogeny and genetic distances

The concatenated sequences included 846 base pairs (bp) for COI and 732 bp for ITS-1. The populations of both *D.circumcisa* and *D.verrucula* showed no variation in either COI or ITS-1.

**Figure 2.**
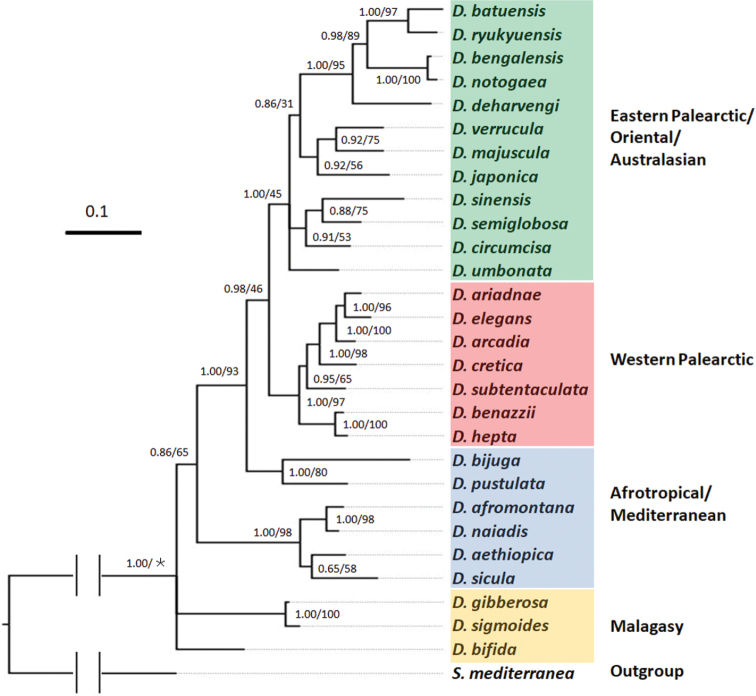
Phylogenetic tree obtained from Bayesian analysis of the concatenated dataset. Numbers at nodes indicate support values (pp/bs).＊: Bootstrap value not applicable to the node, because of different topologies of trees obtained by BI and ML methods. Scale bar: substitutions per site.

The phylogenetic trees obtained by BI and ML from the concatenated dataset showed similar topologies and supported nodes (Fig. 2; Suppl. material 1: Fig. S1). The new species *D.circumcisa* and *D.verrucula* occupy separate branches that are clearly differentiated from their congeners. Notably, *D.circumcisa* is part of a clade consisting of two other species, viz., *D.sinensis* and *D.semiglobosa*, while *D.verrucula* belongs to another clade that includes the species *D.majuscula* and *D.japonica*. These two clades form part of a group of Eastern Palearctic/Oriental/Australasian species that in total comprises four clades of which the interrelationships are unresolved, thus forming a polytomy (Fig. 2). This major clade of Eastern Palearctic/Oriental/Australasian species is molecularly well separated from its sister clade, composed of species from the Western Palearctic.

The separate species status of *D.circumcisa* and *D.verrucula* is supported also by the genetic distances between the species included in our analysis, albeit that COI distances vary greatly among species (Suppl. material 2: Table S1).

The highest distance value between *D.circumcisa* and its congeners is 24.65% (with *D.batuensis* Ball, 1970 and *D.sicula* Lepori, 1948), while the lowest distance value is 11.20% (with *D.umbonata*). With respect to *D.verrucula*, the highest distance value between this species and its congeners is 26.09% (with *D.aethiopica* Stocchino et al., 2002), while the lowest distance value is 15.47% (with *D.umbonata*). Furthermore, there is a 17.15% difference between the two new species.

With respect to ITS-1, *D.circumcisa* and *D.verrucula* show highest distance values with *D.sicula*, which are 18.65% and 17.57%, respectively. Furthermore, their lowest distance values are with *D.majuscula*, which are 4.8% and 2.77%, respectively. For this marker, the molecular distance between the two new species is 4.98% (Suppl. material 3: Table S2).

### Systematic account

#### Order Tricladida Lang, 1884

**Suborder Continenticola Carranza, Littlewood, Clough, Ruiz-Trillo, Baguñà & Riutort, 1998**

**Family Dugesiidae Ball, 1974**

##### Genus *Dugesia* Girard, 1850

###### Dugesia circumcisa

Taxon classificationAnimaliaTricladidaDugesiidae

Chen & Dong sp. nov.

3A8B03EE-2034-58F6-96B5-E06CFBC0FEA8

http://zoobank.org/292AFA17-03F6-4153-BC1D-61F0212F207A

####### Material examined.

***Holotype*.**ZMHNU-YWSZ2, Shiwan Dashan Mountain National Natural Reserve (21°54'34"N, 107°54'52"E), Shangsi County, Guangxi Province, China, alt. 245 m above sea level (a.s.l.), 1 January 2019, coll. G-W Chen, D-Z Dong and co-workers, sagittal sections on 14 slides. ***Paratypes*.**ZMHNU-YWSZ1, ibid., sagittal sections on 28 slides; RMNH VER. 19974.a, ibid., sagittal sections on 26 slides; ZMHNU-YWSZ5, ibid., horizontal sections on 15 slides; ZMHNU-YWSZ6, ibid., transverse sections on 30 slides; ZMHNU-YWSZ8, ibid., sagittal sections on 19 slides; ZMHNU-YWSZ9, ibid., sagittal sections on 15 slides; RMNH VER. 19974.b, ibid., sagittal sections on 21 slides; ZMHNU-YWSZ11, ibid., transverse sections on 60 slides; ZMHNU-YWSZ12, ibid., horizontal sections on 19 slides.

####### Diagnosis.

*Dugesiacircumcisa* is characterised by the presence of the following features: right vas deferens opening at anterior portion of the seminal vesicle, and the left one opening at lateral portion of the vesicle, with the left sperm duct opening dorsally to the right one; two diaphragms in ejaculatory duct, the distal one receiving secretion of penial glands; ejaculatory duct with ventral course through penis papilla and with terminal opening; small nozzle at tip of penis papilla; wide duct connecting male and common atrium; asymmetrical openings of the oviducts into the bursal canal; chromosome complement triploid, with 24 metacentric chromosomes.

####### Etymology.

The specific epithet is derived from the Latin adjective *circumcisus*, ‘pruned of excess, sheared on all sides’, and alludes to the appearance of the tip of the penis papilla.

####### Habitat and reproduction.

**Figure 3.**
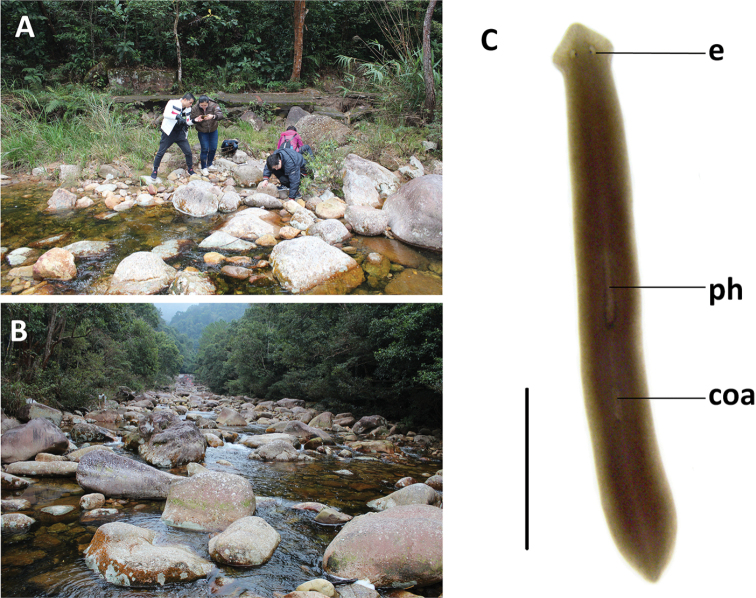
Habitat and external appearance of *Dugesiacircumcisa***A** sampling site **B** habitat **C** sexually mature, live individual. Abbreviations: coa: copulatory apparatus; e: eye; ph: pharynx. Scale bar: 5 mm.

Approximately 20 animals were collected from a freshwater stream on the Shiwan Dashan Mountain (Fig. 3A, B) at an altitude of 245 m a.s.l. (air temperature 8.6 °C, water temperature 12 °C). The Shiwan Dashan Mountain lies in the tropical monsoon forest, which forms a discontinuous system in tropical areas of Asia, Africa, and South America. The most typical tropical monsoon rainforests are distributed in Southeast Asia, with those of mainland China being located mainly near the Tropic of Cancer, thus including parts of the provinces of Hainan, Guangdong, Guangxi, Yunnan, and Tibet. Within this monsoon rainforest of mainland China, Shiwan Dashan Mountain forms its southernmost part. None of the animals was sexually mature at collection. During the first period of 150 days (January to May) in the laboratory culture, the worms only showed asexual reproduction by means of fission. However, during the following days, seven individuals sexualised, while eventually 3/5 of the animals sexualised, although thus far they have not produced any cocoons.

####### Karyology.

**Figure 4.**
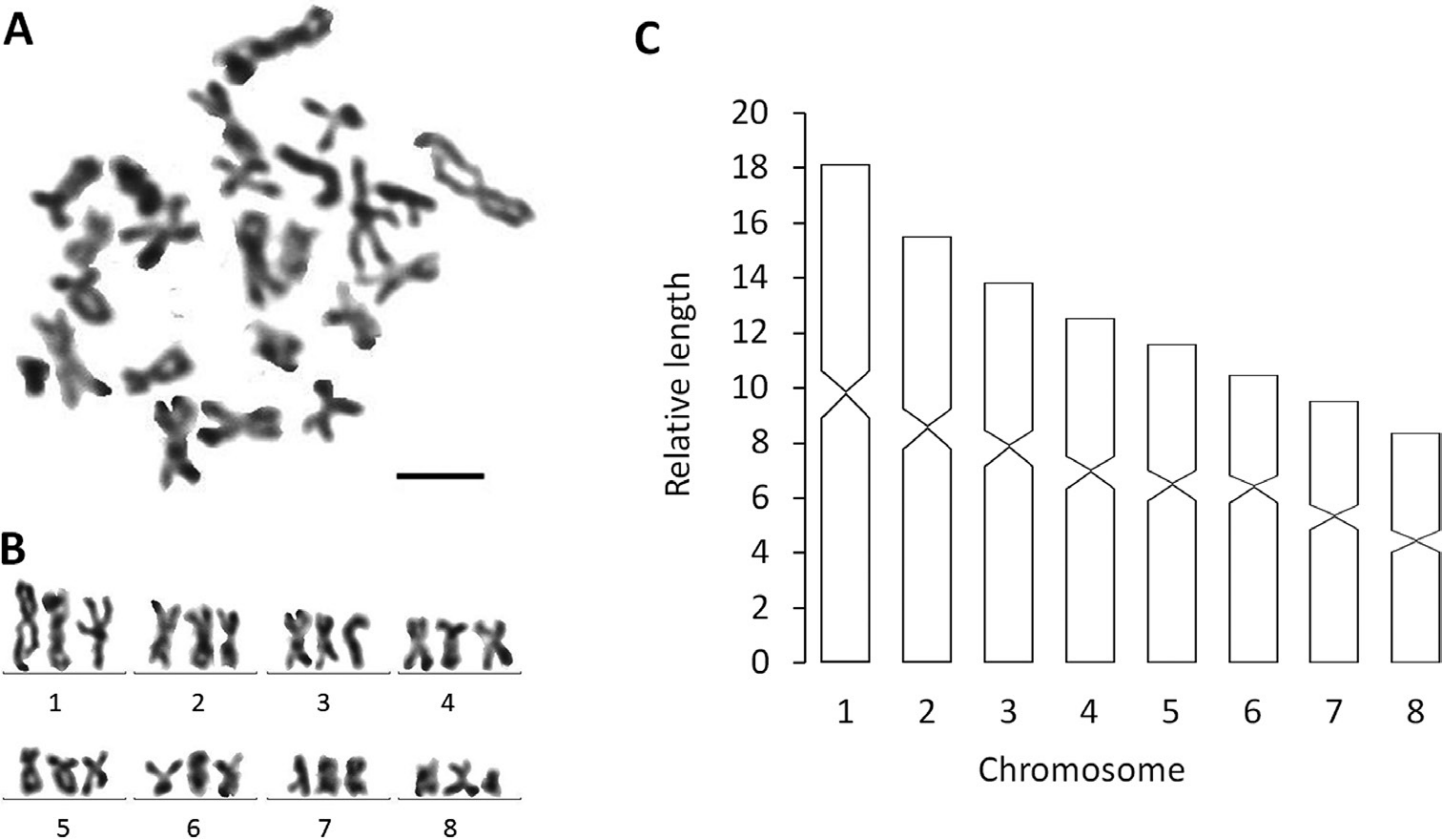
*Dugesiacircumcisa***A** metaphasic plate **B** karyogram **C** idiogram Scale bar: 5 μm.

Each of the five, randomly selected specimens exhibited triploid chromosome complements. In a total of 100 metaphase plates examined, 86 chromosome complements were triploid with 2n = 3× = 24 chromosomes, with all chromosomes being metacentric (Fig. 4); chromosome complements of the remaining 14 plates could not be determined, due to either lack of well-dispersed chromosomes or over-dispersed sets of chromosomes. Karyotype parameters, including relative length, arm ratio, and centromeric index, are given in Table 2. The first pair of chromosomes is clearly larger than the others, being 2.17 times larger than the shortest chromosome.

**Table 2.** Karyotype parameters (mean values and standard deviations) of *Dugesiacircumcisa*; m: metacentric.

Chromosome	Relative length	Arm ratio	Centromeric index	Chromosome type
1	18.15±0.86	1.18±0.12	46.13±2.38	m
2	15.53±0.95	1.23±0.13	45.08±2.44	m
3	13.83±0.15	1.34±0.12	43.01±2.26	m
4	12.53±0.44	1.25±0.11	44.64±2.09	m
5	11.59±0.52	1.29±0.09	44.10±1.67	m
6	10.48±0.14	1.60±0.28	39.22±4.07	m
7	9.55±0.20	1.28±0.16	44.19±2.99	m
8	8.35±0.43	1.15±0.12	46.73±2.39	m

####### Description.

In asexual living specimens, body 8–15 mm in length and 0.8–1.5 mm in width, while in sexualised specimens the body measures 15–22 mm in length and 1.5–2.3 mm in width. Head of low triangular shape and provided with two auricles, as well as two eyes located in pigment-free patches (Fig. 3C). Dorsal surface dark brown, excepting pale body margin and a fuzzy, pale mid-dorsal stripe; accumulations of pigment follow the outline of the pharyngeal pocket. Ventral surface light brown.

**Figure 5.**
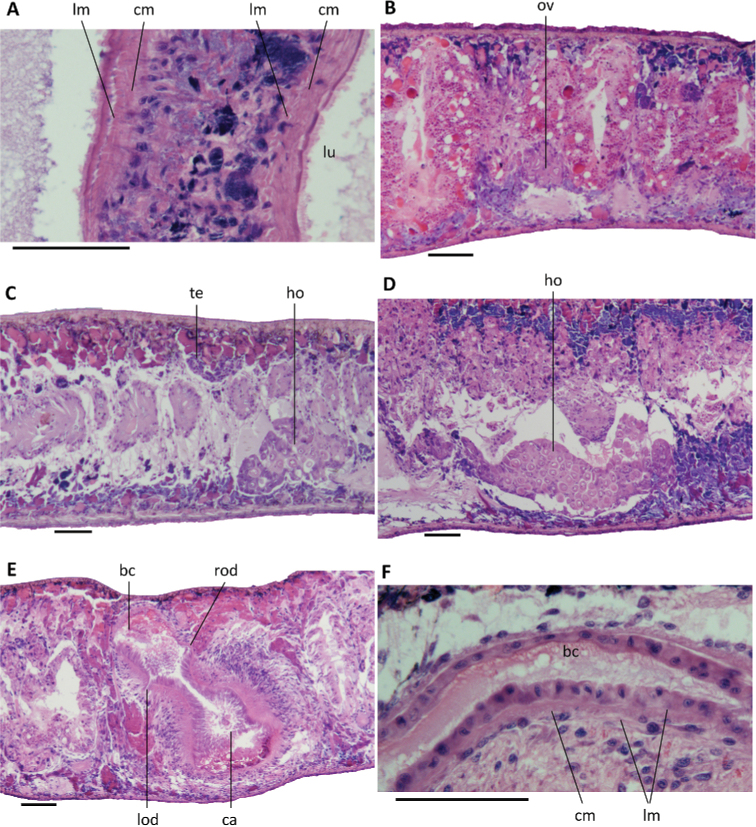
*Dugesiacircumcisa***A** transverse section of pharynx of paratype YWSZ11, showing musculature **B** sagittal section of paratype YWSZ8, showing poorly developed ovary **C** sagittal section of paratype YWSZ9, showing hyperplasic ovaries and poorly developed testes **D** sagittal section of paratype RMNH VER. 19974.a, showing hyperplasic ovaries **E** transverse section of paratype YWSZ11, showing openings of oviducts into bursal canal **F** sagittal section of holotype YWSZ2, showing musculature of bursal canal. Abbreviations: bc: bursal canal; ca: common atrium; cm: circular muscles; ho: hyperplasic ovaries; lm: longitudinal muscles; lod: left oviduct; lu: lumen; ov: ovary; rod: right oviduct; te: testis. Scale bars: 100 μm.

Pharynx situated in the mid-region of the body, measuring ca. 1/5^th^ of the body length (Fig. 3C); mouth opening located at the posterior end of the pharyngeal pocket. Outer pharyngeal musculature composed of a subepidermal layer of longitudinal muscles, followed by a layer of circular muscles (Fig. 5A). Inner pharyngeal musculature composed of a thick subepithelial layer of circular muscles, followed by a thin layer of longitudinal muscles (Fig. 5A).

The ventral ovaries are located at a short distance behind the brain and dorso-medially to the ventral nerve cords. The development of the ovaries differs greatly between specimens. In some animals the ovaries are rather small or even poorly developed (Fig. 5B), while in others the gonads are clearly hyperplasic (Fig. 5C, D). The oviducts arise from the dorsal surface of the ovaries and run dorsally to the ventral nerve cords in a caudal direction. At approximately the level of the gonopore, the oviducts curve dorsad to open separately and asymmetrically into the vaginal portion of the bursal canal, with the right oviduct opening dorsally to the left one (Figs 5E, 7, 8).

**Figure 6.**
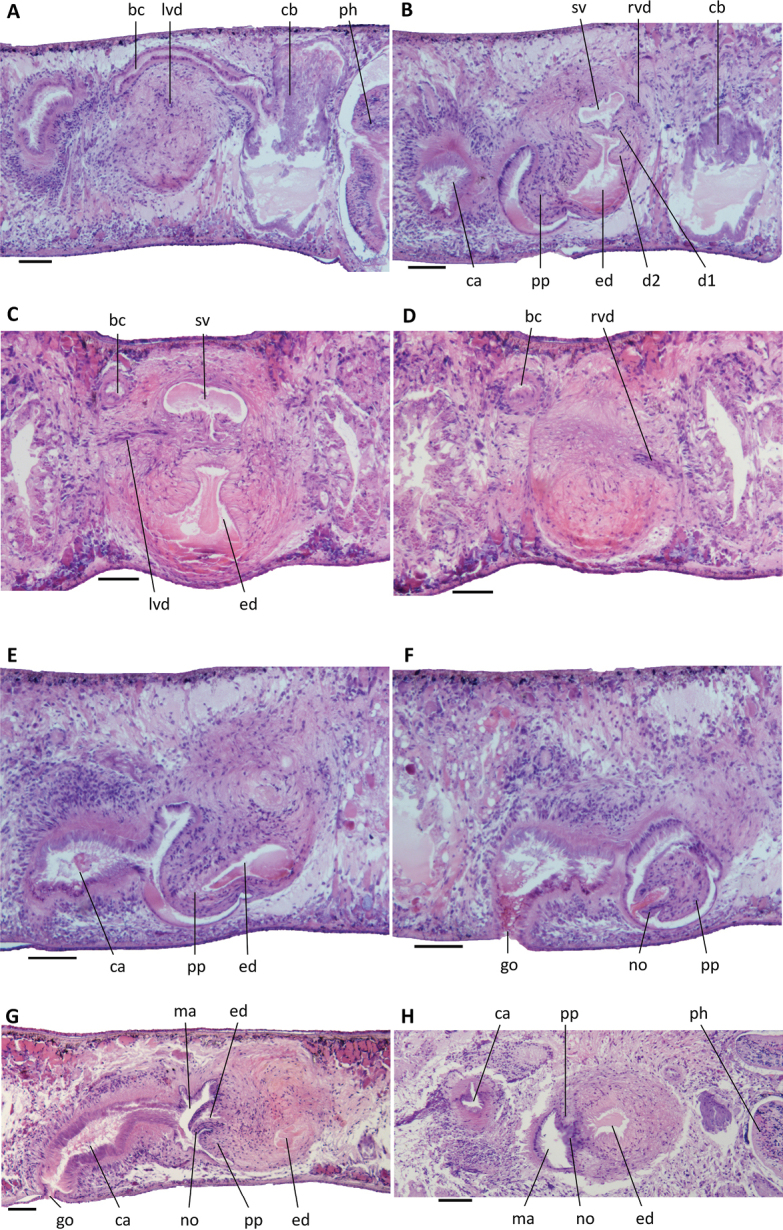
*Dugesiacircumcisa***A** sagittal section of holotype YWSZ2, showing bursal canal and copulatory bursa **B** sagittal section of holotype YWSZ2, showing opening of the right vas deferens into seminal vesicle, and two diaphragms **C** transverse section of paratype YWSZ11, showing left vas deferens, seminal vesicle, and ejaculatory duct **D** transverse section of paratype YWSZ11, showing right vas deferens **E** sagittal section of holotype YWSZ2, showing ejaculatory duct **F** sagittal section of holotype YWSZ2, showing small nozzle at tip of penis papilla **G** sagittal section of paratype YWSZ9, showing small nozzle at tip of penis papilla and the duct connecting male and common atrium **H** horizontal section of paratype YWSZ12, showing small nozzle at tip of penis papilla. Abbreviations: bc: bursal canal; ca: common atrium; cb: copulatory bursa; d: diaphragm; ed: ejaculatory duct; go: gonopore; lvd: left vas deferens; ma: male atrium; no: nozzle; ph: pharynx; pp: penis papilla; rvd: right vas deferens; sv: seminal vesicle. Scale bars: 100 μm.

**Figure 7.**
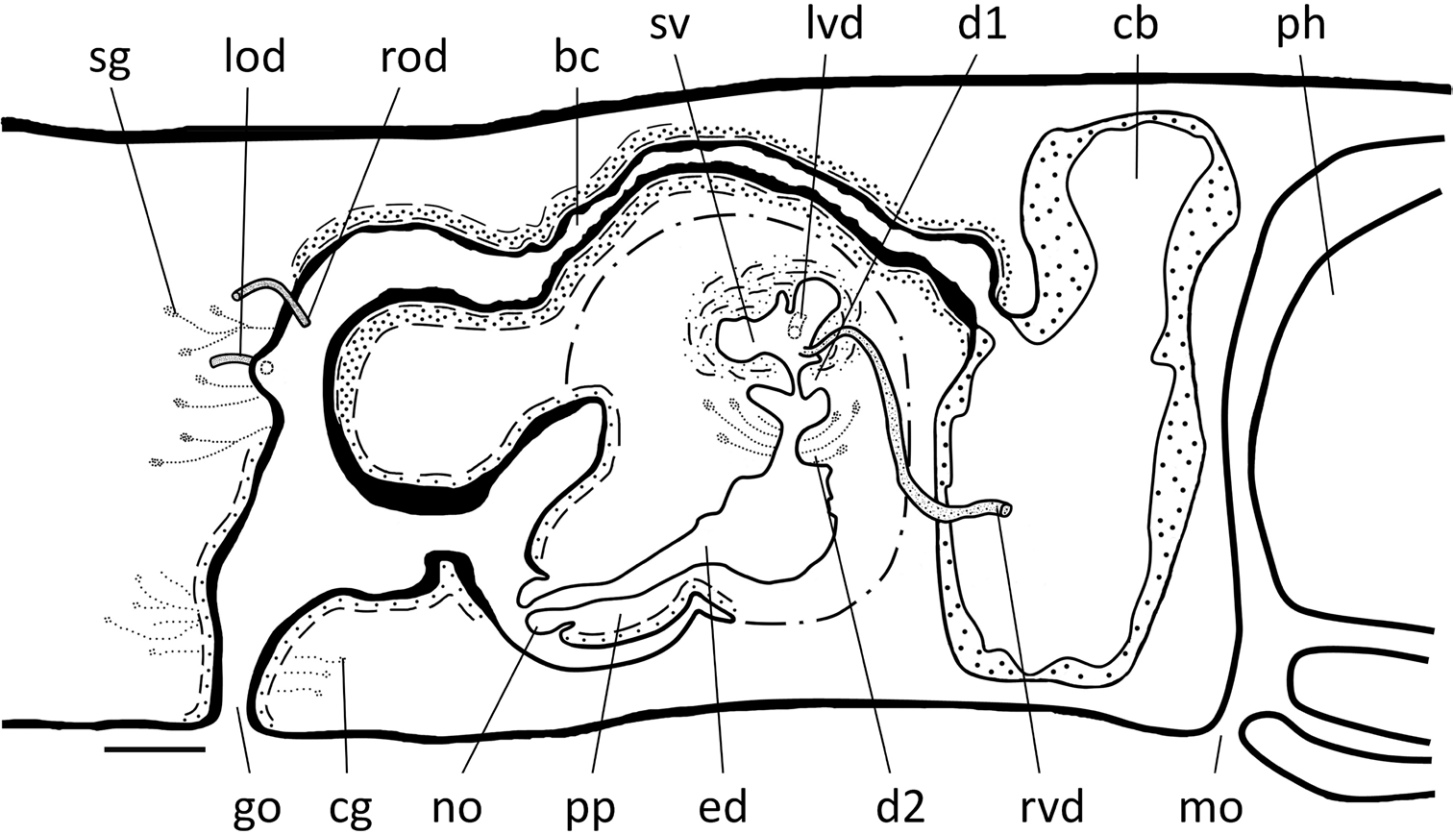
*Dugesiacircumcisa* Sagittal reconstruction of the copulatory apparatus of holotype YWSZ2. Abbreviations: bc: bursal canal; cb: copulatory bursa; cg: cement glands; d1: first diaphragm; d2: second diaphragm; ed: ejaculatory duct; go: gonopore; lod: left oviduct; lvd: left vas deferens; mo: mouth; no: nozzle; ph: pharynx; pp: penis papilla; rod: right oviduct; rvd: right vas deferens; sg: shell glands; sv: seminal vesicle. Scale bar: 100 μm.

**Figure 8.**
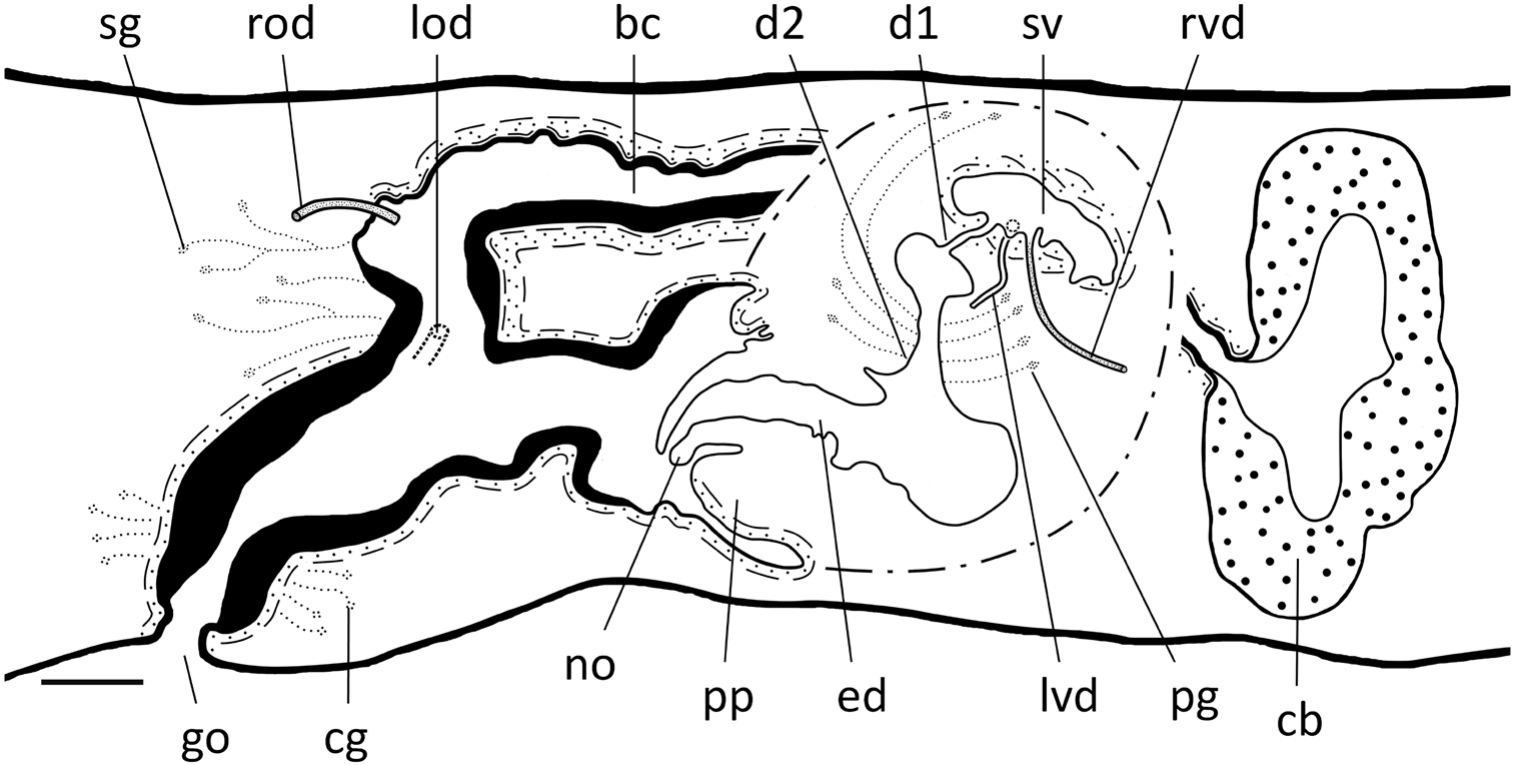
*Dugesiacircumcisa* Sagittal reconstruction of the copulatory apparatus of paratype YWSZ9. Abbreviations: bc: bursal canal; cb: copulatory bursa; cg: cement glands; d1: first diaphragm; d2: second diaphragm; ed: ejaculatory duct; go: gonopore; lod: left oviduct; lvd: left vas deferens; no: nozzle; pg: penial glands; pp: penis papilla; rod: right oviduct; rvd: right vas deferens; sg: shell glands; sv: seminal vesicle. Scale bar: 100 μm.

The large sac-shaped copulatory bursa, which occupies the entire dorso-ventral space, is lined by a vacuolated epithelium with basal nuclei and is almost devoid of any surrounding musculature (Figs 6A, 7, 8). From the postero-dorsal wall of the bursa, the rather narrow bursal canal runs in a caudal direction to the left side of the copulatory apparatus, after which it curves ventrally and opens into the common atrium (Figs 6A, 7, 8). The bursal canal is lined with columnar, nucleated, ciliated cells and surrounded by a thin subepithelial layer of longitudinal muscles, followed by a slightly thicker layer of circular muscle (Fig. 5F). An ectal reinforcement layer of longitudinal muscles runs from the vaginal region to ca. halfway along the bursal canal. Shell glands discharge their erythrophil secretion into the vaginal region of the bursal canal, near the oviducal openings.

The small, dorsally located testes are poorly developed and provided with immature spermatozoa (Fig. 5C). Testicular follicles are arranged on either side of the midline of the body in three or four longitudinal zones, extending from the posterior level of the ovaries to almost the posterior end of the body. Spermatozoa are absent also from the vasa deferentia, which upon reaching the level of the penis bulb curve dorso-mediad and asymmetrically penetrate the wall of the penis bulb. The right sperm duct penetrates the antero-lateral wall of the penis bulb and opens into the anterior portion of the seminal vesicle (Figs 6B, D, 7, 8). The left sperm duct penetrates the lateral wall of the penis bulb and opens through the mid-lateral wall of the seminal vesicle (Figs 6C, 7). Furthermore, the left sperm duct opens dorsally to the right one. The sperm ducts are lined with nucleated cells and are surrounded by a layer of circular muscles.

The large and well-developed penis bulb occupies the major part of the dorso-ventral space and is composed of intermingled muscle fibres (Figs 6B–F, 7, 8). The penis bulb is asymmetrical in that a major portion expands dorsally to well beyond the midline of the body or even almost extends to the dorsal epidermis. This dorsal portion houses the oblate seminal vesicle, which is lined with a flat, nucleated epithelium. Via a valve-like diaphragm in its ventral wall, the seminal vesicle communicates with an expansion or vesicle in the proximal portion of the ejaculatory duct, which has a more or less vertical orientation. Subsequently, this expansion communicates via a large, blunt, valve-like diaphragm with a much larger expansion of the ejaculatory duct (Figs 6B, 7, 8), which is lined by a nucleated epithelium. The second diaphragm receives the openings of erythrophil penis glands, in contrast to the proximal, first diaphragm, which does not receive any secretion.

From the point of the large expansion, the ejaculatory duct changes its vertical orientation and starts to run more or less parallel to the body surface or attains an oblique, ventro-caudal orientation, thus basically conforming to the particular orientation of the penis papilla. The narrow section of the ejaculatory duct that runs between the large expansion and the tip of the penis papilla is lined by an infranucleated epithelium and follows a ventral course through the papilla, opening terminally at its tip (Figs 6F–H, 7, 8). In point of fact, the ejaculatory duct opens at a small nozzle located at the otherwise blunt tip of the penis papilla (Figs 6F, G, 7, 8). This nozzle can either be extended (Figs 6G, 8) or be withdrawn to greater or lesser extent (Fig. 6H).

The asymmetrical penis papilla is covered by an infranucleated epithelium, which is underlain by a subepithelial layer of circular muscle, followed by a layer of longitudinal muscle fibres. The penis papilla almost completely occupies the small male atrium, the latter communicating with the common atrium via a wide duct (Figs 6G, 7, 8). The common atrium opens to the exterior via a gonoduct, which is lined by a columnar epithelium and receives the openings of abundant cement glands.

####### Discussion.

Generally, there is only one diaphragm present in the ejaculatory duct of species of *Dugesia*, and only a few species exhibit two diaphragms, such as *D.bijuga* Harrath & Sluys, 2019, *D.machadoi* de Beauchamp, 1952, *D.mirabilis* de Vries, 1988, *D.maghrebiana*Stocchino et al., 2009, *D.didiaphragma* de Vries, 1988, and *D.semiglobosa* (De Vries 1988; Stocchino et al. 2009; Harrath et al. 2019; Wang et al. 2020). However, in the three first-mentioned species, the ejaculatory duct runs a central course through the penis papilla, in contrast to the ventral trajectories in *D.semiglobosa*, *D.maghrebiana*, *D.didiaphragma*, and the new species *D.circumcisa*. The copulatory bursa of the latter lacks the complex stratified epithelium, which projects through an opening of the bursa that is present in *D.semiglobosa*, while it also lacks the large seminal vesicle enclosed by a highly muscularised, elongated penis bulb as present in *D.didiaphragma* and the knob-like extension on the penis papilla of *D.maghrebiana*.

In species with two diaphragms, the proximal diaphragm is usually small and basically formed by a non-glandular constriction of the seminal vesicle, while the true diaphragm is a larger structure that receives the secretion of penial glands, as is usual for the diaphragm in species of *Dugesia*. The situation in *D.circumcisa* is slightly different in that the proximal diaphragm is not small but consists of a well-developed valve.

Generally, in species of *Dugesia* the openings of the left and right sperm ducts into the intrabulbar seminal vesicle are located at ca. the same level. However, in *D.circumcisa* the vasa deferentia open asymmetrically into the seminal vesicle. Such asymmetrical openings have been reported explicitly for *D.bifida* Stocchino & Sluys, 2014, in which the sperm ducts open halfway into the vesicle, with the right duct opening dorsally to the left one. This contrasts with the situation in *D.circumcisa*, in which the right sperm duct opens into the anterior portion of the seminal vesicle and the left duct opens through the mid-lateral wall of the seminal vesicle, with the left sperm duct opening dorsally to the right one.

The characteristic nozzle at the tip of the penis papilla in *D.circumcisa* is paralleled in *D.bakurianica* Porfirjeva, 1958, *D.bijuga*, and perhaps also in *D.sinensis*. From that perspective, it is interesting that *D.bijuga* also possesses two diaphragms (see above). However, other parts of the male copulatory apparatus of *D.circumcisa* are different from that of *D.bijuga* (e.g., glands opening at a major portion of the blunt penis papilla, as well as atrial folds in the latter species). Furthermore, in the phylogenetric tree, *D.circumcisa* is far removed from *D.bijuga* (Fig. 2). *Dugesiacircumcisa* differs from *D.sinensis* in the presence of atrial folds in the latter, while it differs from *D.bakurianica* in that this species only has a single diaphragm.

Another characteristic feature of *D.circumcisa* is the rather wide and long duct connecting the male atrium with the common atrium. Generally, in species of *Dugesia* the male atrium opens more or less directly into the common atrium, without interpolation of a well-defined duct. Apart from *D.circumcisa*, other exceptions to this ground-plan condition can be found in *D.bactriana* de Beauchamp, 1959, *D.bengalensis* Kawakatsu, 1983, *D.bifida*, *D.capensis* Sluys, 2007, and *D.colapha* Dahm, 1967. However, other characters prevent synonymisation of *D.circumcisa* with any of these species, which in the case of *D.bifida* is supported also by completely different positions in the phylogenetic tree (Fig. 2).

###### Dugesia verrucula

Taxon classificationAnimaliaTricladidaDugesiidae

Chen & Dong sp. nov.

FD13C0AB-5AD2-5368-BDBF-57BA9CA4AF06

http://zoobank.org/3DC3C5B0-1846-4A5D-9E41-04FD869447A4

####### Material examined.

***Holotype*.**ZMHNU-ZJYA5, sagittal sections on 27 slides, Shiwan Dashan Mountain National Natural Reserve (21°53'40"N, 107°54'30"E; alt. 520 m a.s.l.), Shangsi County, Guangxi Province, China, 1 January 2019, coll. G-W Chen, D-Z Dong and co-workers. ***Paratypes*.**ZMHNU-ZJYA1, ibid., sagittal sections on 25 slides; ZMHNU-ZJYA2, ibid., sagittal sections on 36 slides; ZMHNU-ZJYA3, ibid., sagittal sections on 38 slides; RMNH VER. 19975.a, ibid., sagittal sections on 34 slides; ZMHNU-ZJYA6, ibid., horizontal sections on 24 slides; ZMHNU-ZJYA7, ibid., transverse sections on 30 slides; RMNH VER. 19975.b, ibid., sagittal sections on 24 slides; ZMHNU-ZJYA9, ibid., horizontal sections on 19 slides; ZMHNU-ZJYA10, ibid., sagittal sections on 32 slides; ZMHNU-ZJYA11, ibid., sagittal sections on 30 slides.

####### Diagnosis.

*Dugesiaverrucula* is characterised by the presence of the following features: large size of the live worm, usually exceeding 3.5 cm in length; asymmetrical openings of the oviducts into the bursal canal; subterminal opening of the ventrally displaced ejaculatory duct; vasa deferentia symmetrically opening into the postero-lateral portion of the seminal vesicle; well-developed duct between seminal vesicle and diaphragm; single dorsal bump near root of penis papilla; bursal canal with pleated wall and spacious posterior section; unstalked cocoons; chromosome complement diploid with 16 metacentric chromosomes.

####### Etymology.

The specific epithet is derived from the Latin *verrucula*, small wart, and alludes to the dorsal bump on the penis papilla; the specific epithet is treated as an adjective.

####### Habitat and reproduction.

**Figure 9.**
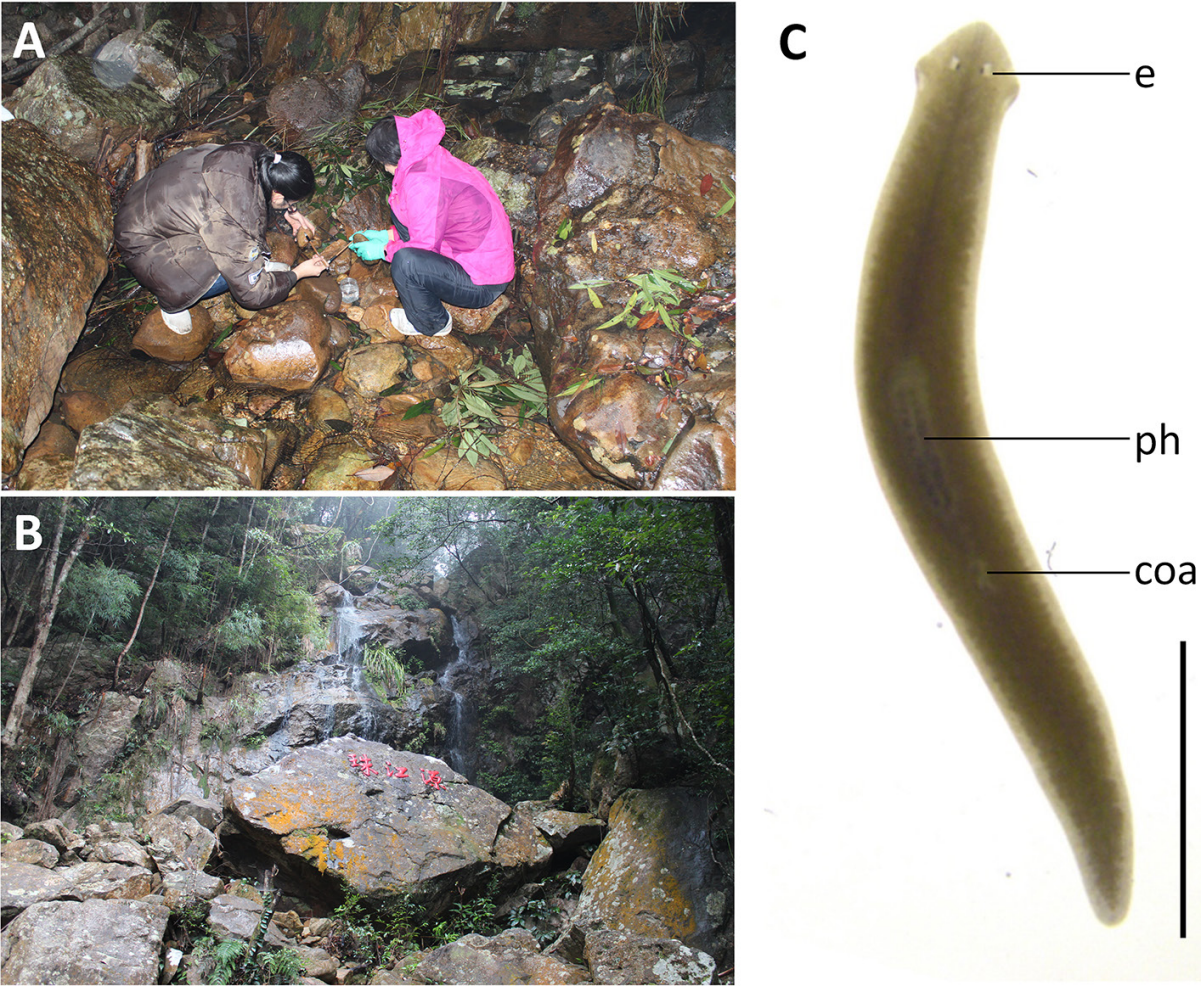
Habitat and external appearance of *Dugesiaverrucula***A** sampling site **B** habitat **C** sexually mature living individual (ca. 50 days old). Abbreviations: coa: copulatory apparatus; e: eye; ph: pharynx. Scale bar: 5 mm.

The average annual temperature of the tropical monsoon rain forest in the Shiwan Dashan Mountain National Natural Reserve ranges between 20 and 25 °C, while in the coldest month (January), the average temperature is ca. 10–13 °C. The worms were collected from a pond under a waterfall on the Shiwan Dashan Mountain, with a water temperature of 8.4 °C, while air temperature was 5.5 °C (Fig. 9A, B). It is noteworthy that the water temperature is lower than the coldest average temperature of the tropical monsoon rain forest and that, thus, the worms live under relatively harsh climatic conditions, as compared to that of the forest itself. At collection, ten specimens were sexually mature. After ca. 30 days of culturing under laboratory conditions, the animals produced unstalked spherical cocoons (approx. 1.5 mm in diameter) that were firmly attached to the glass wall of the containers. Newly laid cocoons at first were red but turned dark brown after two or three days. After 10–20 days, 8–11 juveniles hatched from each cocoon. Juvenile planarians were light brown, after ca. 2 days, measuring 1.5–2.0 mm in length and 0.18–0.21 mm in width. After approximately 50 days, the animals measured 15–20 mm in length and 1.4–1.8 mm in width and were sexual (Fig. 9C). Fission was not observed in the laboratory culture.

####### Karyology.

**Figure 10.**
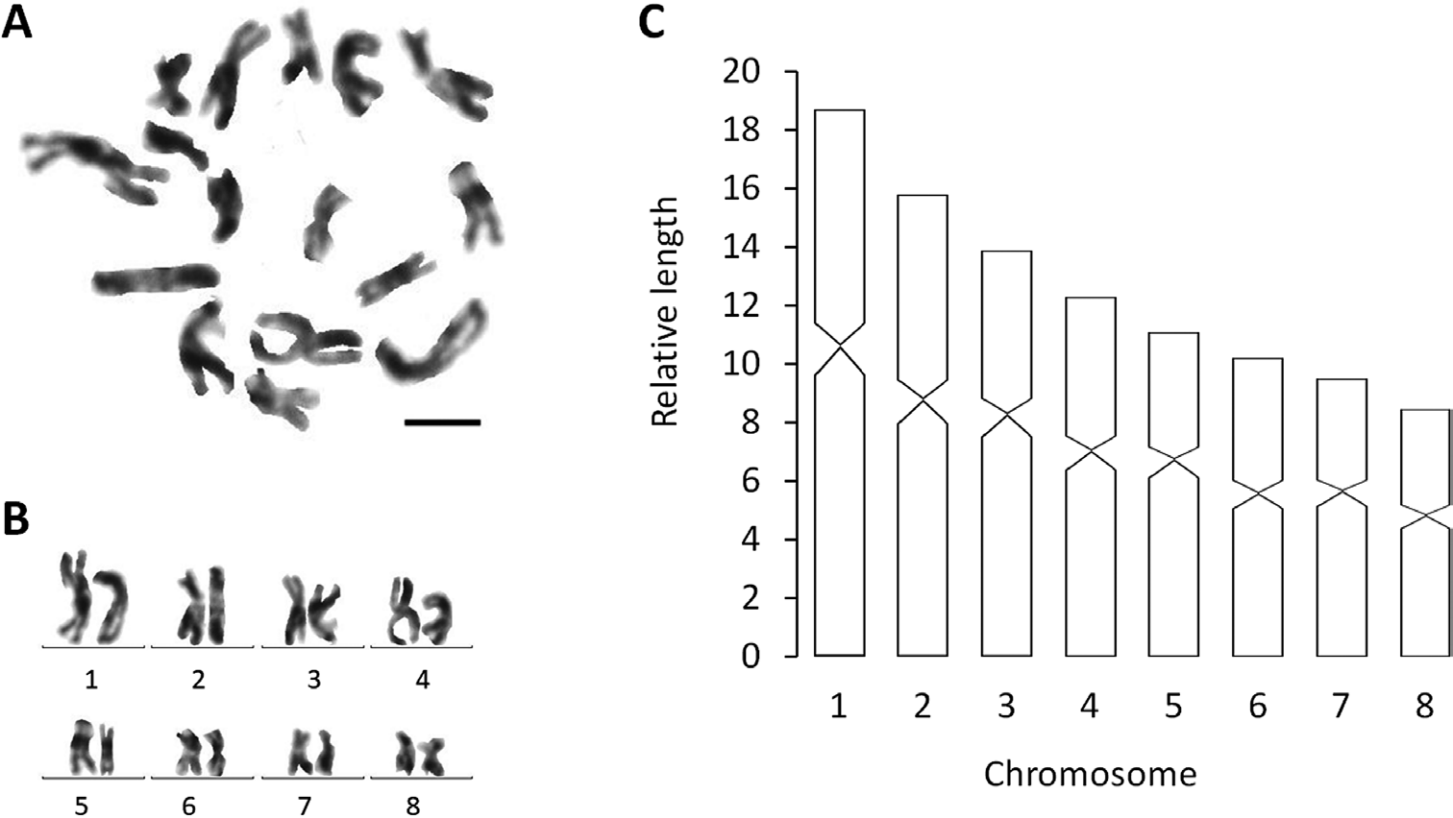
*Dugesiaverrucula***A** metaphasic plate, **B** karyogram **C** idiogram. Scale bar: 5 μm.

Each of the six, randomly selected specimens exhibited diploid chromosome complements. In a total of 100 metaphase plates examined, 85 chromosome complements were diploid with 2n = 2× = 16 chromosomes, with all chromosomes being metacentric (Fig. 4); chromosome complements of the remaining 15 plates could not be determined, due to either lack of well-dispersed chromosomes or over-dispersed sets of chromosomes. The first pair of chromosomes is clearly larger than others, being 2.21 times larger than the shortest chromosome. Karyotype parameters, including relative length, arm ratio, and centromeric index are given in Table 3, while a chromosomal plate and idiogram are shown in Fig. 10.

**Table 3.** Karyotype parameters (mean values and standard deviations) of *Dugesiaverrucula*; m: metacentric.

Chromosome	Relative length	Arm ratio	Centromeric index	Chromosome type
1	18.72±0.66	1.31±0.16	43.54±3.00	m
2	15.81±0.56	1.25±0.12	44.75±2.62	m
3	13.88±0.42	1.47±0.27	41.35±3.36	m
4	12.31±0.29	1.34±0.15	43.03±2.63	m
5	11.1±0.33	1.59±0.30	39.89±4.45	m
6	10.22±0.46	1.19±0.09	45.81±1.87	m
7	9.49±0.32	1.48±0.25	41.49±3.28	m
8	8.47±0.53	1.32±0.18	43.36±3.22	m

####### Description.

The body of live, sexual specimens measures 2.8–3.9 mm in length and 1.8–2.8 mm in width. Low-triangular head provided with two blunt auricles and two eyes, which are located in the centre of the head and placed in pigment-free spots and house numerous retinal cells (Fig. 9C). Dorsal surface brown, excepting the pale body margin and an accumulation of pigment following the outline of the pharynx. Furthermore, there is a dark brown mid-dorsal stripe, extending from the head to the posterior end of the body. The ventral surface is light brown.

**Figure 11.**
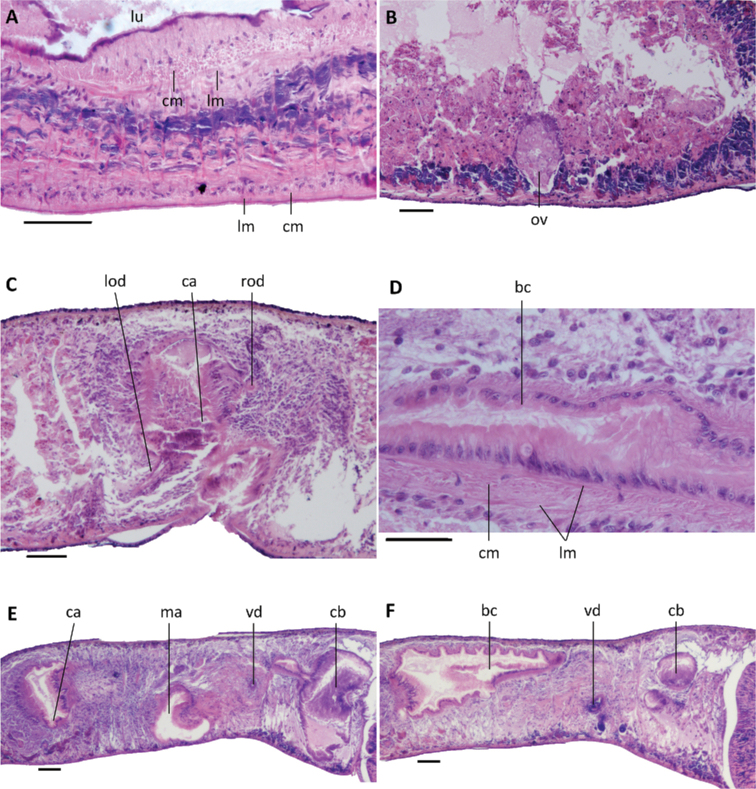
*Dugesiaverrucula***A** sagittal section of paratype RMNH VER. 19975.a, showing the musculature of the pharynx **B** sagittal section paratype RMNH VER. 19975.a, showing an ovary **C** transverse section of paratype ZJYA7, showing asymmetrical openings of the oviducts **D** sagittal section of paratype ZJYA11, showing musculature of bursal canal **E** sagittal section of paratype ZJYA11, showing copulatory bursa **F** sagittal section of paratype ZJYA11, showing wide portion of bursal canal, with its pleated walls. Abbreviations: bc: bursal canal; ca: common atrium; cb: copulatory bursa; cm: circular muscles; lm: longitudinal muscles; lod: left oviduct; lu: lumen; ma: male atrium; ov: ovary; rod: right oviduct; vd: vas deferens. Scale bars: 100 μm.

Pharynx situated in the mid-region of the body and measuring ca. 1/5^th^ of the body length (Fig. 9C). Mouth opening located at the posterior end of the pharyngeal pocket. Outer pharyngeal musculature composed of a subepidermal layer of longitudinal muscles, followed by a layer of circular muscles; inner pharyngeal musculature composed of a thick subepithelial layer of circular muscles, followed by a layer of longitudinal muscles (Fig. 11A).

Ventral ovaries located at a short distance behind the brain, occupying ca. 1/6^th^ of the dorso-ventral space (Fig. 11B). The oviducts run ventrally in a caudal direction posteriorly to the genital pore, after which they curve dorsally to open separately and asymmetrically into the ventral portion of the bursal canal. The right branch opens dorsally to the left one, the latter actually opening into the common atrium (Figs 11C, 13, 14).

**Figure 12.**
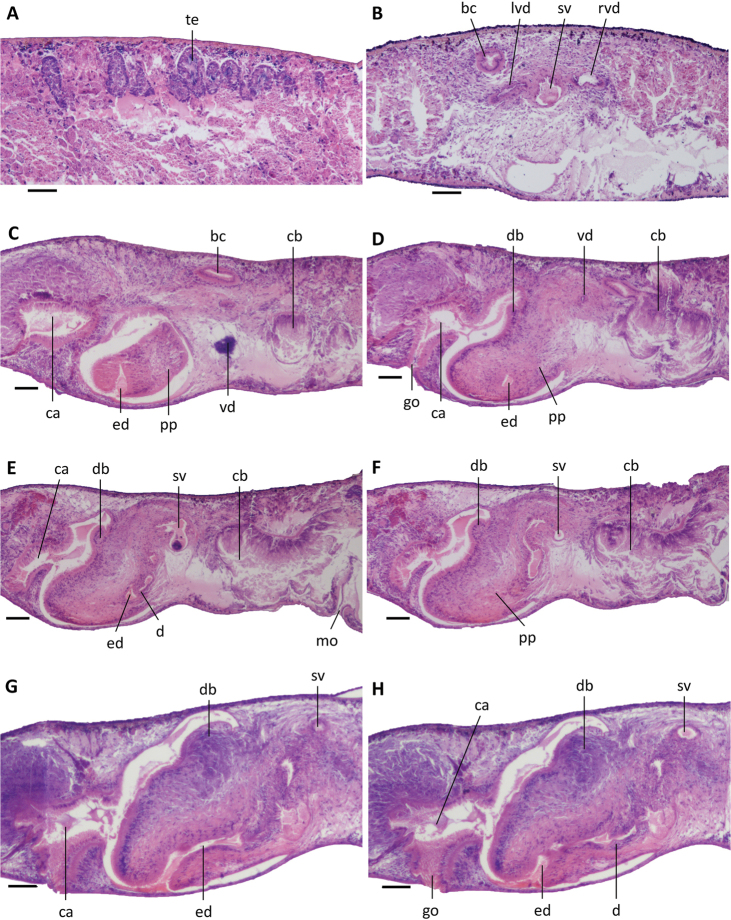
*Dugesiaverrucula***A** sagittal section of holotype ZJYA5, showing the testes **B** transverse section of paratype ZJYA7, showing vasa deferentia and seminal vesicle **C** sagittal section of holotype ZJYA5, showing subterminal opening of ejaculatory duct at tip of penis papilla **D** sagittal section of holotype ZJYA5, showing dorsal bump and copulatory bursa **E** sagittal section of holotype ZJYA5, showing seminal vesicle, diaphragm in the ejaculatory duct, dorsal bump, and copulatory bursa **F** sagittal section of holotype ZJYA5, showing seminal vesicle, dorsal bump, and copulatory bursa **G** sagittal section of paratype ZJYA11, showing subterminal opening of ejaculatory duct and dorsal bump **H** sagittal section of paratype ZJYA11, showing gonopore, ejaculatory duct, and diaphragm. Abbreviations: bc: bursal canal; ca: common atrium; cb: copulatory bursa; d: diaphragm; db: dorsal bump; ed: ejaculatory duct; go: gonopore; lvd: left vas deferens; mo: mouth; pp: penis papilla; rvd: right vas deferens; sv: seminal vesicle; te: testis; vd: vas deferens. Scale bars: 100 μm.

**Figure 13.**
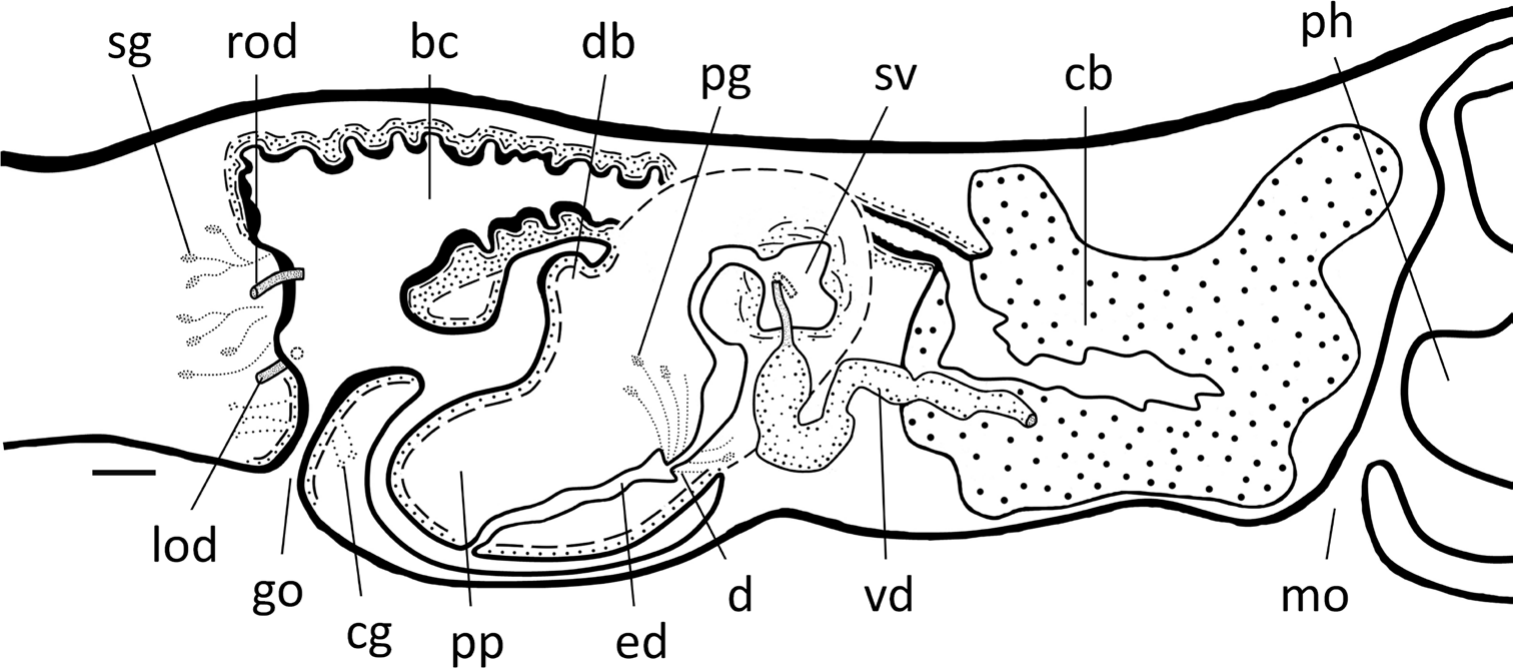
*Dugesiaverrucula* Sagittal reconstruction of the copulatory apparatus of holotype ZJYA5. Abbreviations: bc: bursal canal; cb: copulatory bursa; cg: cement glands; d: diaphragm; db: dorsal bump; ed: ejaculatory duct; go: gonopore; lod: left oviduct; mo: mouth; pg: penial glands; ph: pharynx; pp: penis papilla; rod: right oviduct; sg: shell glands; sv: seminal vesicle; vd: vas deferens. Scale bar: 100 μm.

**Figure 14.**
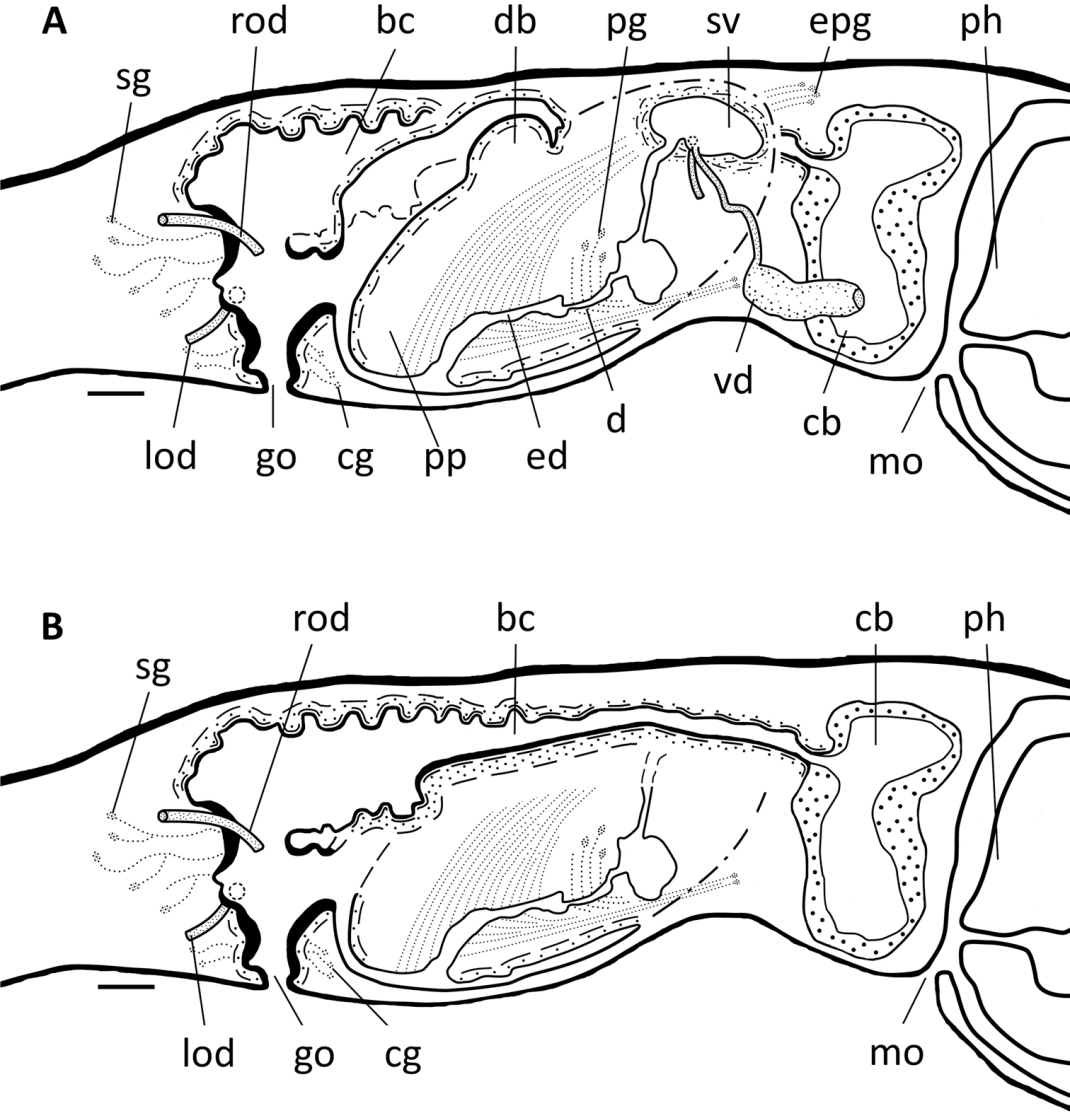
*Dugesiaverrucula* Sagittal reconstruction of the copulatory apparatus of paratype ZJYA11 **A** sagittal reconstruction of male copulatory apparatus **B** sagittal reconstruction of female copulatory apparatus. Abbreviations: bc: bursal canal; cb: copulatory bursa; cg: cement glands; d: diaphragm; db: dorsal bump; ed: ejaculatory duct; epg: extrabulbar penial glands; go: gonopore; lod: left oviduct; mo: mouth; pg: penial glands; ph: pharynx; pp: penis papilla; rod: right oviduct; sg: shell glands; sv: seminal vesicle; vd: vas deferens. Scale bar: 100 μm.

The large, sac-shaped copulatory bursa occupies the entire dorso-ventral space, and is lined by a vacuolated epithelium with basal nuclei (Fig. 11E, F). From the dorso-posterior wall of the bursa, the bursal canal runs in a caudal direction to the left side of the copulatory apparatus. The anterior section of the bursal canal is narrow, but dorsally to the male copulatory apparatus the canal expands in dorso-ventral direction and also presents a distinctly folded wall, formed by numerous pleats, particularly in its dorsal wall. In particular the posterior portion of the bursal canal expands to form a spacious chamber that almost imperceptibly grades into the equally spacious common atrium (Figs 11D, 13, 14B). The ventral portion of the latter communicates with the gonoduct (Figs 12D, G, 13, 14B).

The bursal canal is lined with columnar, nucleated, ciliated cells and is surrounded by a thin subepithelial layer of longitudinal muscles, followed by a thicker layer of circular muscle. An ectal reinforcement layer of longitudinal muscles runs from the vaginal region to ca. halfway along the bursal canal (Figs 11D, 13, 14B). Shell glands discharge their erythrophil secretion into the vaginal region of the bursal canal, near the oviducal openings.

The well-developed testes are situated dorsally and provided with mature spermatozoa (Fig. 12A). On either side of the midline of the body, testicular follicles are arranged in five or six longitudinal zones and extend from the posterior level of the ovaries to almost the posterior end of the body.

The vasa deferentia expand to form large spermiducal vesicles, filled with sperm (Figs 12C, 13, 14A). At the level of the penis bulb, the sperm ducts curve medio-dorsad and considerably decrease in diameter before penetrating the lateral wall of the penis bulb, and, subsequently, separately and symmetrically open into the ventro-lateral or mid-lateral portion of the relatively large, rounded seminal vesicle (Figs 12B, 13, 14A). The latter is lined by a flat, nucleated epithelium and is surrounded by a layer of intermingled muscle fibres.

The ejaculatory duct arises from the antero-dorsal wall of the seminal vesicle and, subsequently, curves downwards towards the ventral root of the penis papilla. Near the ventral root of the papilla the ejaculatory duct is provided with a small diaphragm and changes its more or less vertical orientation by turning towards the tip of the penis papilla, having a subterminal opening at its tip (Figs 12C–H, 13, 14A). The ejaculatory duct is lined by an infranucleated epithelium and is hardly surrounded by any musculature. The diaphragm receives the secretion of erythrophil glands, while the ejaculatory duct also receives the abundant secretion of extrabulbar penis glands (Figs 12E–H, 14A).

Because of the ventrally displaced course of the ejaculatory duct, the penis papilla is asymmetrical, with its dorsal lip being considerably larger than the ventral lip. The conical or sub-cylindrical penis papilla has an oblique, ventro-caudad orientation and is covered by an infranucleated epithelium, which is underlain by a subepithelial layer of circular muscle, followed by a layer of longitudinal muscle fibres. Near its dorsal root, the penis papilla is provided with a pronounced bump (Figs 12D–H, 13, 14A).

####### Discussion.

The most characteristic feature of *D.verrucula* is the permanent dorsal bump on its penis papilla, a similar character being known only from *Dugesiagibberosa* Stocchino & Sluys, 2017. However, the latter is provided with two dorsal bumps on its penis papilla instead of one, while its ejaculatory duct opens terminally at the tip of the papilla (Stocchino et al. 2017), in contrast to the subterminal opening in *D.verrucula*.

Apart from the penial bump and the subterminal opening of the ejaculatory duct, other characteristic features of *D.verrucula* are the asymmetrical penis papilla with ventrally displaced ejaculatory duct (character 1, state 1 in Sluys et al. 1998; see also Stocchino et al. 2017), and the presence of a duct between the seminal vesicle and the diaphragm (character 5, state 1 in Sluys et al. 1998). Besides *D.verrucula*, these two character states are also expressed in the following species: *D.andamanensis* (Kaburaki, 1925), *D.austroasiatica* Kawakatsu, 1985, *D.batuensis*, *D.bengalensis*, *D.borneana* Kawakatsu, 1972, *D.burmaensis* (Kaburaki, 1918), *D.deharvengi* Kawakatsu & Mitchell, 1989, *D.gibberosa*, *D.hymanae* (Sivickis, 1928), *D.indonesiana* Kawakatsu, 1973, *D.japonica*, *D.lindbergi* De Beauchamp, 1959, *D.mertoni* (Steinmann, 1914), *D.naiadis* Sluys, 2013, *D.notogaea* Sluys & Kawakatsu, 1998, *D.novaguineana* Kawakatsu, 1976, *D.ryukyuensis* Kawakatsu, 1976, *D.siamana* Kawakatsu, 1980, *D.tamilensis* Kawakatsu, 1980, *D.majuscula*, *D.umbonata* (see Sluys et al. 1998; Sluys et al. 2013; Stocchino et al. 2017; Song et al. 2020; Wang et al. 2021). However, these species all differ from *D.verrucula* in the gross anatomy of the copulatory apparatus or in detailed character states, such as the openings of the oviducts (common oviduct or symmetrical openings, in contrast to the asymmetrical oviducal openings in *D.verrucula*), opening of the ejaculatory duct at the tip of the penis papilla (terminal, in contrast to the subterminal opening in *D.verrucula*), or presence of a penial valve (absent in *D.verrucula*).It is noteworthy that the cocoons of *D.verrucula* are unstalked, since usually in species of *Dugesia* the egg capsules are provided with a pedicel (Sluys and Riutort 2018), the only other exception being *D.bifida* from Madagascar (Stocchino et al. 2014). However, in other aspects of their reproduction *D.verrucula* and *D.bifida* are rather different, in that in the latter cocoons were produced by ex-fissiparous, sexualised specimens, which had developed hyperplasic ovaries; the juveniles emerging from these fertile cocoons gave rise to new fissiparous clones. This reproductive strategy differs much from the fully sexual life cycle of *D.verrucula*.

## General discussion

Inter-specific genetic distances of both COI and ITS-1 reveal that *D.circumcisa* and *D.verrucula* are well-separated from their congeners. The lowest COI distance values between *D.circumcisa* and *D.verrucula* and other congeners are 11.20% and 15.47%, respectively, while the distance between the two new species is 17.15% (Suppl. material 2: Table S1). With respect to ITS-1, the lowest distance values between *D.circumcisa* and *D.verrucula* and other congeners are 4.8% and 2.77%, respectively, while the distance between the two new species is 4.98% (Suppl. material 3: Table S2). Previous studies showed that the lowest COI distance value between species usually is in the range between 6% and 10% (Làzaro et al. 2009; Solà et al. 2013; Stocchino et al. 2017; Harrath et al. 2019). For ITS-1, the lowest distances reported between species ranged between 1% and 7% (Làzaro et al. 2009; Solà et al. 2013; Stocchino et al. 2017). All of these values are surpassed by the genetic distances determined for both *D.circumcisa* and *D.verrucula*, which thus support the results of the morphological and phylogenetic analyses.

The topology of our phylogenetic tree (Fig. 2) basically agrees with results from previous phylogenetic analyses (Lázaro et al. 2009; Solà et al. 2013; Stocchino et al. 2017; Song et al. 2020). It is noteworthy that although the two new species occur at the same geographic location, they belong to two different clades, together with other species from southern China.

The two new species show a haploid number of n = 8 metacentric chromosomes, thus conforming to the situation that in the genus *Dugesia* the basic chromosome number is 7, 8 or 9 (Stocchino et al. 2004). Chromosome portraits similar to the ones of *D.circumcisa* and *D.verrucula* are also present in the following species: many Sardinian populations of *D.benazzii* Lepori, 1951, *D.etruscalabronica* Lepori, 1950, *D.elegans* de Vries, 1984, *D.gonocephala* (Dugès, 1830), *D.japonica*, *D.indonesiana*, *D.majuscula*, *D.sagitta* (Schmidt, 1861), *D.semiglobosa*, *D.salina* (Whitehouse, 1914), and presumably also *D.colapha* Dahm, 1967 (cf. Dahm 1967; Benazzi and Gourbault 1975; Kawakatsu et al. 1976; Ball 1979; De Vries 1984; Deri et al. 1999; Pala et al. 1999; Stocchino 2018; Wang et al. 2020; Bromley-Schnur 2021).

The number of 8–11 juveniles hatching from a single cocoon of *D.verrucula* falls at the higher end of the range as reported for other sexual species of *Dugesia*, such as *D.benazzii* (8–10 hatchlings), *D.etrusca* (8–10), *D.hepta* Pala et al., 1981 (8–10), *D.cretica* (Meixner, 1928) (4–15) (Bromley 1974; Kobayashi et al. 2009; Stocchino and Manconi 2013). However, in some species of *Dugesia*, ex-fissiparous individuals are also able to produce fertile cocoons, for example in *D.sicula* (1 or 2 hatchlings), *D.afromontana* Stocchino & Sluys, 2012 (1 or 2), *D.arabica* Harrath & Sluys, 2013 (1–3), *D.ryukyuensis* (1–5), and *D.aethiopica* (1–6) (Harrath et al. 2013; Stocchino and Manconi 2013 and references therein; Stocchino et al. 2014). The number of young hatching from such capsules produced by ex-fissiparous specimens often is lower than in sexual species.

In contrast, *D.circumcisa* never produced cocoons and only showed asexual reproduction by means of fission, which corresponds with its poorly developed or hyperplasic ovaries and the triploid chromosome complement. It has been established that in such abnormal ovaries the oocytes are anomalous (Harrath et al. 2014), thus preventing regular oogenesis.

## Supplementary Material

XML Treatment for Dugesia circumcisa

XML Treatment for Dugesia verrucula

## Supplementary materials

Supplementary material 1 Figure S1 Data type Phylogenetic tree Explanation note Phylogenetic tree obtained from ML analysis of the concatenated dataset. Numbers at nodes indicate support values (bootstrap). Scale bar: substitutions per site. https://binary.pensoft.net/file/586167This dataset is made available under the Open Database License (http://opendatacommons.org/licenses/odbl/1.0/). The Open Database License (ODbL) is a license agreement intended to allow users to freely share, modify, and use this Dataset while maintaining this same freedom for others, provided that the original source and author(s) are credited.Lei Wang, Jin-zi Chen, Zi-mei Dong, Guang-wen Chen, Ronald Sluys, De-zeng Liu

Supplementary material 2 Table S1 Data type Genetic distances Explanation note Genetic distances for COI. Highest and lowest distance values between the two new Chinese species and Oriental-Australasian congeners indicated in red and blue, respectively. Purple: distance value between the two new species. https://binary.pensoft.net/file/586168This dataset is made available under the Open Database License (http://opendatacommons.org/licenses/odbl/1.0/). The Open Database License (ODbL) is a license agreement intended to allow users to freely share, modify, and use this Dataset while maintaining this same freedom for others, provided that the original source and author(s) are credited.Lei Wang, Jin-zi Chen, Zi-mei Dong, Guang-wen Chen, Ronald Sluys, De-zeng Liu

Supplementary material 3 Table S2 Data type Genetic distances Explanation note Genetic distances for ITS-1. Highest and lowest distance values between the two new Chinese species and Oriental-Australasian congeners indicated in red and blue, respectively. Purple: distance value between the two new species. https://binary.pensoft.net/file/586169This dataset is made available under the Open Database License (http://opendatacommons.org/licenses/odbl/1.0/). The Open Database License (ODbL) is a license agreement intended to allow users to freely share, modify, and use this Dataset while maintaining this same freedom for others, provided that the original source and author(s) are credited.Lei Wang, Jin-zi Chen, Zi-mei Dong, Guang-wen Chen, Ronald Sluys, De-zeng Liu
