# Supplementary figures and images for: Two new species of Dugesia (Platyhelminthes, Tricladida, Dugesiidae) from the tropical monsoon forest in southern China
Source: Zookeys. 2021 Sep 8;1059:89–116. doi: 10.3897/zookeys.1059.65633 (PMC8443540; doi:10.3897/zookeys.1059.65633)

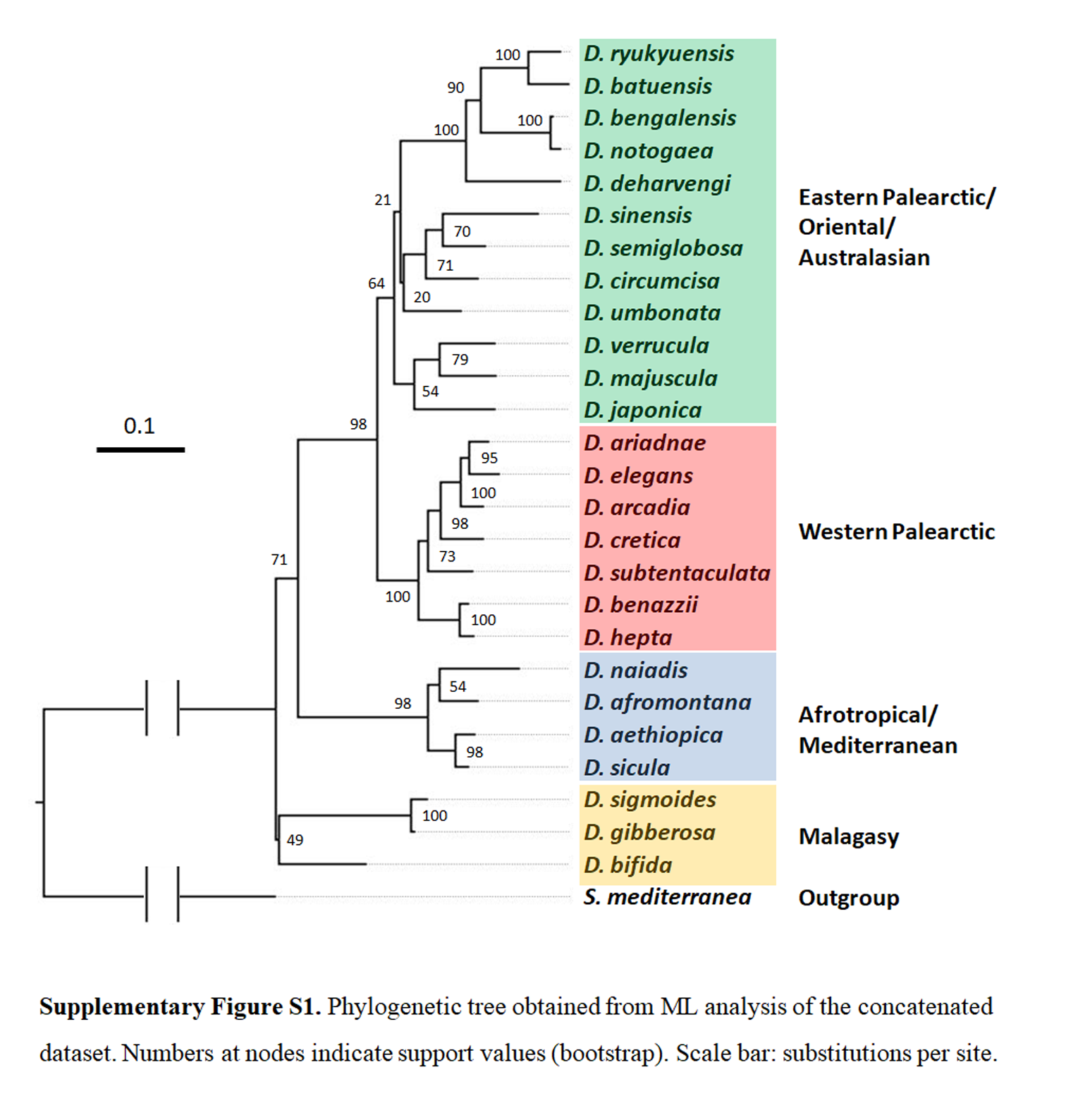

Supplement: Supplementary material 1 — Figure S1 [file zookeys-1059-089-s001.tif]
